# A pragmatic randomized controlled trial of standard care versus corticosteroids plus standard care for treatment of pneumonia in adults admitted to Kenyan hospitals (SONIA)

**DOI:** 10.12688/wellcomeopenres.18401.2

**Published:** 2025-05-28

**Authors:** Ruth Lucinde, Abdirahman Abdi, Benedict Orindi, Stella Mwakio, Henry Gathuri, Edwin Onyango, Salome Chira, Morris Ogero, Lynda Isaaka, Jimmy Shangala, Irene Njeri Oginga, Alvin Wachira, Evans Manuthu, Hazel Kariuki, Jared Nyikuli, Cyprian Wekesa, Amos Otedo, Hannah Bosire, Steve Biko Okoth, Winston Ongalo, David Mukabi, Wilber Lusamba, Beatrice Muthui, Nicholas Kirui, Isaac Adembesa, Caroline Mithi, Mohammed Sood, Nadia Ahmed, Bernard Gituma, Vera Bina Ongaki, Matiko Giabe, Charles Omondi, Loice Achieng Ombajo, Wangeci Kagucia, Mike English, Mainga Hamaluba, Lynette Isabella Ochola-Oyier, Dorcas Kamuya, Philip Bejon, Ambrose Agweyu, Samuel Akech, Anthony Oliwa Etyang

**Affiliations:** 1 KEMRI-Wellcome Trust Research Programme (KWTRP) Centre for Geographic Medical Research – Coast (CGMRC), Kilifi, Kenya; 2 Medicine, Kiambu Level Five Hospital, Kiambu, Kenya; 3 Medicine, Machakos Level Five Hospital, Machakos, Kenya; 4 Medical, Kitale County Referral Hospital, Kitale, Kenya; 5 Medical, Naivasha Level Five Hospital, Naivasha, Kenya; 6 Medical, Bungoma County Referral Hospital, Bungoma, Kenya; 7 Medical, Kisumu County Hospital, Kisumu, Kenya; 8 Medical, Kakamega County General Hospital, Kakamega, Kenya; 9 Medical, Busia County Referral Hospital, Busia, Kenya; 10 Medical, Mama Lucy Kibaki Hospital, Nairobi, Kenya; 11 Medical, Moi Teaching and Referral Hospital, Eldoret, Kenya; 12 Medical, Kenyatta University Teaching and Referral Hospital, Kiambu, Kenya; 13 Medical, Coast General Teaching and Referral Hospital, Mombasa, Kenya; 14 Medical, Kilifi County Hospital, Kilifi, Kenya; 15 Medical, Mbagathi County Hospital, Nairobi, Kenya; 16 Medical, Kisii Teaching and Referral Hospital, Kisii, Kenya; 17 Medical, Jaramogi Oginga Odinga Teaching and Referral Hospital, Kisumu, Kenya; 18 Infectious Diseases, The University of Nairobi, Nairobi, Kenya; 19 Medical, Kenyatta National Hospital, Nairobi, Kenya

**Keywords:** Pragmatic, Randomized-Controlled Trial, Steroids, Pneumonia, Adults, Mortality, Africa

## Abstract

**Background:**

Mortality among adults admitted to hospital with community acquired pneumonia in resource-limited settings is high. Recent studies conducted in high-income settings have demonstrated beneficial effects of low-dose corticosteroids in reducing mortality in patients with severe community acquired pneumonia. It is unknown whether these findings apply to low-income settings such as sub-Saharan Africa.

This pragmatic randomized-controlled open-label trial will determine the effect of adjunctive low-dose corticosteroids in the management of adults admitted to hospital with community acquired pneumonia on mortality 30-days post-randomization.

**Methods:**

We will enroll and randomize 2180 patients admitted with a diagnosis of community acquired pneumonia into two arms: the control and intervention arm. Those in the control arm will receive standard care for the treatment of community acquired pneumonia i.e., combination therapy with a beta-lactam and macrolide antibiotic. Those in the intervention arm will receive up to 10-days treatment with low-dose oral corticosteroids in addition to standard care. All participants will be followed up to 30- days post randomization and their final status recorded (alive or dead).

**Discussion:**

If adjunctive low-dose oral corticosteroids are found to be beneficial, this easily scalable intervention would significantly reduce the currently high mortality associated with community acquired pneumonia.

Pan-African Clinical Trials Registry: PACTR202111481740832; ISRCTRN registry: ISRCTN36138594

## Introduction

### Background and rationale {6a}

Community acquired pneumonia (CAP) is a common cause of illness and death among adults in Kenya and other low and middle-income countries (LMICs) ^1^. The pattern of CAP in sub-Saharan Africa differs from that in other regions due to higher prevalences of coinfections like pulmonary tuberculosis and HIV and differences in risk factors for severe disease in this population compared to others ^2^.

Inflammation plays an important role in the pathophysiology of pneumonia with elevated levels of inflammatory cytokines and infiltration of interstitial lung tissue with various inflammatory cells being observed in patients with severe disease ^3^. Dysregulated inflammation results in a sepsis-like syndrome with eventual organ dysfunction in severe cases ^4^.

Corticosteroids have a role in improving patients outcomes in CAP by modulating the release of inflammatory mediators such as cytokine and reducing pulmonary inflammation ^5^. While previous data on the effect of corticosteroids in the management of CAP have been inconclusive ^6– 9^, data from the RECOVERY trial during the COVID-19 pandemic ^10^ and other studies such as the ESCAPe ^11^ and CAPE COD ^12^ trials have demonstrated a beneficial effect of adding corticosteroids to the management of patients admitted with severe CAP. Consequently, several systematic reviews and metanalyses now report corticosteroids have beneficial effects in reducing progression to invasive ventilation, duration of hospital stay and mortality ^13– 16^.

These studies are however potentially not applicable to settings such as Kenya for the following reasons; a) mortality in this setting is ~30%, which is much higher than that observed in studies in high income settings, b) patients in our setting are younger (35–50 years) compared to average age of 65 years in other settings c) the prevalence of comorbidities (e.g. HIV and TB) in patients with CAP in LMIC settings is different. Without evidence from a sub-Saharan African population, questions remain on the effectiveness of adjunctive corticosteroids in the management of patients admitted with severe CAP in the region.

 We are conducting a pragmatic randomized controlled open-label clinical trial to test if there is a survival benefit in the use of adjunctive low-dose corticosteroid therapy in patients admitted to hospital with CAP.

## Objectives {7}

Primary Objectives

- To determine if there is a difference in the proportion of patients with community acquired pneumonia that die within 30 days of being randomized to receive adjunctive corticosteroid treatment compared to those that are randomized to receive standard treatment only

Secondary Objectives

- To determine if there is a difference in the proportion of patients with community acquired pneumonia that die within seven, 14 and 21 days of being randomized to receive adjunctive corticosteroid treatment compared to those that are randomized to receive standard treatment only
- To determine if there is a difference in the proportion of patients with community acquired pneumonia that die in hospital and out of hospital after being randomized to receive adjunctive corticosteroid treatment or standard treatment for community acquired pneumonia only
- To determine the correlation of pre-existing and treatment induced changes in the participants’ immune and metabolic profiles with study outcomes by trial arm

## Trial design {8}

We will conduct a pragmatic open label randomized controlled clinical trial. Eligible participants will be recruited from the in-patient adult medical wards of the participating hospitals. Patients admitted to the general wards as well as the High Dependency Units (HDU) and Intensive Care Units (ICU) will be eligible to join the trial and will be identified by the attending clinician and a study clinician.

Participants enrolled into the trial will be randomized 1:1 to either receive standard of care for CAP or standard of care plus a 10-day course of low-dose oral corticosteroids. A subset of all recruited participants, about 50 per trial arm, will be further randomized to have a blood draw at four timepoints during their participation. For these participants, a 10 ml blood sample will be collected at enrollment, 24, 48 and 72 hours after enrollment for an immunology sub study.

All participants will be followed up to their 30 ^th^ day after enrollment to determine their vital status. The median duration of hospital admission for patients with CAP in the participating hospitals is 4 days, therefore having the primary outcome ascertained at 30 days is unlikely to result in misclassification of patients where some are recorded as being alive only to die a few days later.

Being a pragmatic trial, the study will not interfere with any procedure, investigations, or treatments that the attending clinicians administer to participants. However, all treatments received will be recorded.

## Methods: Participants, interventions and outcomes

### Study setting {9}

The proposed study will be conducted within the Clinical Information Network (CIN), and five other hospitals. The CIN is a collaborative effort between KEMRI-Wellcome Trust Research Programme, Kenya Paediatric Association (KPA), Kenya’s Ministry of Health (MoH), and several county hospitals ^17– 19^. CIN, which commenced in 2013, initially sought to improve the care provided to in-patient children and sick new-borns through improving the collection and use of information obtained from routine medical records. At the onset of the COVID-19 pandemic, it expanded to the adult wards to conduct surveillance on severe acute respiratory illness (SARI) and now includes collection of routine data from adult wards. A total of 24 hospitals are currently part of the CIN. Use of de-identified routine data obtained from the CIN hospitals for observational research has been approved by the Kenya Medical Research Institute Scientific and Ethics Review Unit (KEMRI SERU).

**Figure 1.**
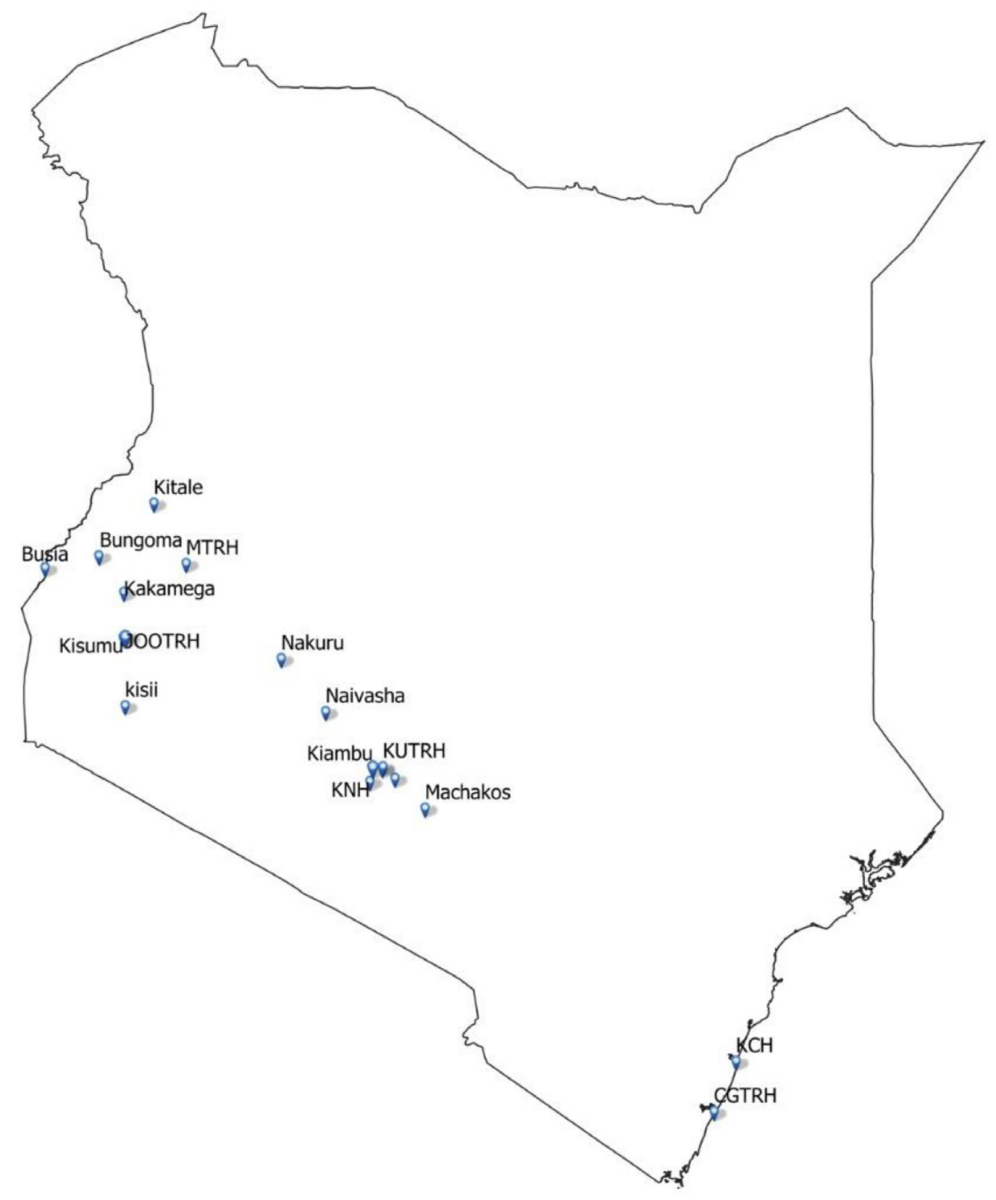
Map of Kenya showing distribution of SONIA trial sites. This figure represents the distribution of SONIA trial sites. Eight sites are located in western Kenya, eight around central Kenya and two are at the Kenyan coast.

The trial will recruit participants at the following 15 hospitals which are part of CIN: Bungoma County Referral Hospital, Busia County Referral Hospital, Embu Level 5 Teaching and Referral Hospital, HomaBay County Referral Hospital, Jaramogi Oginga Odinga Teaching and Referral Hospital, Kakamega County General Hospital, Kiambu Level 5 Hospital, Kisii Teaching and Referral Hospital, Kisumu County Hospital, Kitale County Referral Hospital, Machakos Level 5 Hospital, Mama Lucy Kibaki Hospital, Mbagathi County Hospital, Naivasha Level 5 Hospital and Nakuru County Referral Hospital ( Figure 1). Five other hospitals that are trial sites but are not part of CIN include Coast General Teaching and Referral Hospital, Kenyatta National Hospital, Kenyatta University Teaching and Referral Hospital, Kilifi County Hospital, and Moi Teaching and Referral Hospital, Eldoret ( Figure 1).

### Eligibility criteria {10}

We will recruit patients who have been admitted to hospital with a diagnosis of community acquired pneumonia and for whom COVID-19 results are either negative or unknown. Diagnosis of CAP will be based on clinical criteria recommended by the world health organization (WHO) ^20, 21^ and described in detail in the inclusion criteria below. Unless there are specific reasons justifying exclusion from the study as detailed in section on inclusion and exclusion criteria, we will not exclude patients which conditions such as HIV, diabetes mellitus or other presenting conditions except those indicated below.

### Inclusion criteria

- Adults aged 18 years or over
- Admitted to hospital with a diagnosis of community-acquired pneumonia. Pneumonia will be based on a clinical definition as follows: the presence of at least 2 of the following signs and symptoms for less than 14 days: cough, fever, dyspnea, hemoptysis, chest pain or crackles on chest examination ^20, 21^.
- Admitted to hospital within the previous 48 hours in this current illness
- Provides written informed consent

### Exclusion criteria

- Diagnosis of COVID-19 confirmed via polymerase chain reaction (PCR) of nasopharyngeal/oropharyngeal (NP/OP) swabs or antigen rapid diagnostic tests (RDTs) done at the hospital. This criterion only applies if the test result is known at the point of enrollment
- Hospital acquired pneumonia- defined as pneumonia in a patient who has been in hospital for >48 hours who did not have the symptoms at admission ^21^
- Known or suspected condition which in the opinion of attending clinician requires treatment with corticosteroids, including but not limited to chronic obstructive pulmonary disease, asthma, adrenal insufficiency, Pneumocystis Jirovecii pneumonia (PCP).
- If the clinician strongly suspects COVID-19 and wants to provide corticosteroids to the patient because of this suspicion, then the patient will be excluded from the trial.
- Pregnancy or breast feeding
- Any contraindication to corticosteroid administration

### Who will take informed consent? {26a}

The trial has a study clinician (non-degree trained, referred to as clinical officer in Kenya) posted to each of the hospitals, who will recruit participants, act as a local facilitator to reinforce compliance with the study protocol, and also obtain informed consent. The clinical officer will seek written informed consent from all eligible patients. Deferred written informed consent will be sought from patients if the patient is too unwell to provide consent within the first 48-hours of admission. This process, commonly implemented in clinical trials taking place in emergency situations ^6^, entails a short verbal assent process from legally authorized representatives (LARs) at the time of enrolment by a trained study staff followed later by full written consent after the patient’s condition improves (within 48 hours of recruitment). The information given during this verbal assent process includes key elements of the International Conference on Harmonization Good Clinical Practice (ICH-GCP) recommendations. For patients who die prior to the obtaining full written consent, assent will be used as permission to be included in the trial and use of their data. A copy of the consent form and participant information sheet (PIS) will be provided to the patient or LAR.

### Additional consent provisions for collection and use of participant data and biological specimens {26b}

Written informed consent will be sought from all participants for the use of their data and blood samples, where applicable, for future studies.

## Interventions

### Explanation for the choice of comparators {6b}

The intervention, adjunctive low-dose corticosteroids, is being compared to the current standard of care treatment. While a placebo control could have been used, this would be difficult to implement in the context of a pragmatic trial. Having a mortality end point mitigates against any shortcomings arising from the lack of a placebo control group.

### Intervention description {11a}

Trial participants will be randomized 1:1 to either of the two trial arms; standard care for community acquired pneumonia alone (control arm) or standard care plus a 10-day course of low-dose oral corticosteroids (intervention arm).

Participants randomized to the control arm will receive treatment according to local and WHO guidelines for CAP which currently recommend a beta lactam antibiotic and a macrolide for 7–14 days ^22^. In order to minimize variability in the standard treatment offered at the different hospitals, the study team will provide hard copies of the treatment guidelines, as we have demonstrated that this is associated with better adherence ^23^. In the event of stockouts of standard treatment, the trial will procure the medications for study patients as prescribed by the treating physician, and in accordance with trial treatment allocation. No further interventions will be made in this pragmatic clinical trial.

Participants who are randomized to the intervention arm will receive the standard care treatment for CAP as outlined above and any of the equivalent doses of corticosteroids listed in Table 1 for a period of 10 days. The study will provide the corticosteroids for all participants in the intervention arm based on local availability. The corticosteroids will be administered orally, including via nasogastric tube for patients on ventilation or otherwise unable to take the medications orally.

**Table 1.** Corticosteroids to be used in the trial. This table details the corticosteroids being used as the trial intervention. The corticosteroid doses represented in the table are bioequivalent.

Corticosteroid	Once daily dose
Dexamethasone	6mg
Betamethasone	5mg
Hydrocortisone	160mg
Methylprednisolone	30mg
Prednisolone	50mg
Prednisone	50mg

All other care will be determined by the clinical team primarily responsible for the patient’s care.

### Criteria for discontinuing or modifying allocated interventions {11b}

During participation, the trial intervention may be stopped, and the participant withdrawn for the following reasons:

- The participant’s attending physician may withdraw the study drug in those randomized to the intervention arm due to side effect/s or if the corticosteroids seem to cause worsening of a participant’s existing condition(s). Additionally, they may recommend administration of corticosteroids to a participant randomized to the control arm if in their opinion such a participant requires a corticosteroid as part of their care. Changes in treatment and reasons for change will be captured into the database in such cases
- If a participant tests positive for COVID-19 after joining the trial and undergoing randomization, they will be withdrawn from the trial. This is because there will no longer be meeting the eligibility criteria, in addition to loss of equipoise since they will require to be on corticosteroids.
- If a participant requests to be withdrawn from the trial and was in the intervention arm, the corticosteroids will be discontinued and the participant withdrawn from the trial.

### Strategies to improve adherence to interventions {11c}

While in-patient, trial staff will review all participants daily and ensure they are adherent to their prescribed medication, both standard care and corticosteroids where relevant. At discharge, participants will be advised to complete their medication as prescribed. Because of the pragmatic nature of the trial, trial staff will not conduct any procedures to monitor and/or improve adherence to treatment after discharge.

### Relevant concomitant care permitted or prohibited during the trial {11d}

There is no specific concomitant care prohibited during participation in the trial. All of the care for the participants will be as determined by the treating physicians.

Participants enrolled into the trial will not be permitted to co-enrol in other interventional studies during their 30-days of participation. Participation in studies that do not involve an intervention, that is, observational studies, is acceptable.

### Provisions for post-trial care {30}

The trial intervention, low-dose oral corticosteroids, have in been in clinical use for a long time. At the doses being given to trial participants, they are not likely to cause long-term harm to participants. If a participant reports a suspected unexpected serious adverse event (SUSAR) that is deemed to be causally related to their participation in the trial, the trial will provide care for the participant until resolution of symptoms. Such cases will be reported to ethics and regulatory committees as well as the sponsor.

### Outcomes {12}

The primary outcome for the trial will be mortality at 30 days after randomization. This will be recorded as ‘dead or alive’.

Secondary outcomes will be:

- Mortality at 7,14- and 21-days following randomization
- In-hospital mortality compared to mortality after discharge from hospital (up to 30 days post randomization)
- Correlation of pre-existing and treatment induced changes in the participants’ immune and metabolic profiles with study outcomes

Other descriptive results will be described such as duration of hospital stay, admission to ICU/referral to higher level hospital and serious adverse events as reported by the clinical teams and patients on follow-up phone calls, bearing in mind the pragmatic nature of the trial.

### Participant timeline {13}

All participants enrolled in the trial will be reviewed once daily while in-patient by a member of the study team. During these reviews, data on clinical progress will be recorded. The data collected will include documentation of antibiotics and other treatments that the patient is receiving.

After discharge, a member of the study team will make a phone call to the participant or their next of kin (where a participant is not reachable on their personal phone) on day 14 and day 30 post randomization to find out how the patient is doing including whether readmitted to a hospital, fully recovered or if the participant has died.

During the telephone follow-up, data on the results of any additional tests performed and/or medications received after the hospitalization will also be collected. If the patient is reported to be deceased, the date of death and cause of death (where available) will be recorded.

There will be no further follow up after day 30 post randomization into the study.

### Sample size {14}

The primary hypothesis is that adjunct corticosteroid treatment will reduce mortality in the intervention arm by ~25% (relative measure). This is a conservative measure as the studies described in the background report relative risk reductions of between 33% and 61%. Observational data indicates 30% in-hospital mortality among based on data collected from CIN hospitals in the past one year and Kilifi County Hospital over a period of 5 years ^24^. We have made a conservative assumption that 30-day mortality in the control arm of the trial will be 20% and that 30-day mortality in the intervention arm will be 15%. Allowing for 5% loss to follow-up, a sample size of 1,090 patients per study arm (total 2,180 patients, ~ 390 deaths) will achieve statistical power of 85%, with a 2-sided type 1 error rate of 5%.

### Recruitment {15}

Study clinicians will work with admitting clinicians at the outpatient departments, admissions area in the ward, and nursing teams at the medical wards to ensure they are informed of all patients admitted with respiratory illness. They will then screen these patients for eligibility for the trial and enrol those who provide written informed consent. A window period of 48 hours between admission and recruitment will be implemented to allow study clinicians adequate time to identify all eligible participants ( Table 2).

**Table 2.** Summary of trial activities. This table summarises trial activities and procedures being conducted once a participant is recruited into the trial.

Study Day	Day of recruitment (Day 0)	During Hospitalization	After hospital discharge
Confirm eligibility	x		
Obtain written informed consent	x		
Randomization	x		
Patient issued with study number and a random treatment allocation	x		
Study Intervention (Standard care alone or standard care plus corticosteroid)	x	Daily for up to 10 days ^a^	x ^a^
Follow-up phone call			Day 14 Day 30
Data Collection	x	x	x
Adverse events		x	x
Blood samples ^b^	x	x ^b^	

^a^ If patient is discharged before 10 days, they will continue with corticosteroid medication as an outpatient until day 10. ^b^ Blood samples will only be collected from 100 randomly selected participants (50 in each arm) on day 0,1,2 and 3

## Assignment of interventions: allocation

### Sequence generation {16a}

Once eligibility has been confirmed and informed consent provided, all patients will be randomized to receive either:

a) Standard of care, OR,

b) Standard of care plus adjunctive low-dose corticosteroids for 10 days

The random allocation sequence will be computer-generated at the KEMRI-Wellcome Trust Research Programme according to a block randomization list of random block sizes for each site.

### Concealment mechanism {16b}

At each site, printed and sealed sequential randomization cards will be used for treatment allocation. The envelopes will be numbered in a sequential order to prevent out of order randomization and each study site will have a unique set of randomization cards numbered from the first to the last.

### Implementation {16c}

The site clinician will conduct randomization using the next numbered sealed opaque envelope. To avoid out-of-order treatment allocation due to bias, the randomization number, enrollment number and time of enrollment of each participant will be counterchecked with a master list during ad hoc data checks.

Blinding of allocation and treatment outcome will not be possible in this open label trial. This limitation is however mitigated by the objective nature of mortality as a primary outcome.

## Assignment of interventions: Blinding

### Who will be blinded {17a}

This is an open-label pragmatic trial and blinding is not relevant.

### Procedure for unblinding if needed {17b}

This is an open-label pragmatic trial and blinding is not relevant.

## Data collection and management

### Plans for assessment and collection of outcomes {18a}

Trial staff will be trained on data quality and the necessary data to be collected for trial participants. Basic sociodemographic and clinical data for the participants will be collected from their medical records while in the hospital.

The trial staff will not conduct additional medical assessments but will record findings from assessment done the attending clinical team.

The main trial outcome is vital status (dead or alive) at 30 days after enrolment. Outcome data will be collected via phone call if the participant will have been discharged from the hospital. For participants who are still in-patient by the 30 ^th^ day of participation, outcome data will be recorded on the same day in-person.

Data will be abstracted on to electronic case report forms (eCRFs). Regular data checks will be conducted on the database to ensure completeness of the data.

### Plans to promote participant retention and complete follow-up {18b}

The study clinician will review participants daily while in-patient to ensure adherence to prescribed medication and solicit for adverse events. After discharge, participants will be contacted on their 14 ^th^ and 30 ^th^ day of participation. To do this, at least three different contact details for each participant will be collected. Additionally, the study team will record dates and frequency of visits to regular clinics at the study site e.g., cardiac clinic, diabetes clinic etc., that the participant attends to help with tracing.

Participants will be encouraged to reach out to the study team at any time during their participation if they have health related concerns or questions.

### Data management {19}

Data will be directly entered into electronic CRFs in an electronic database. The database is compliant with Good Clinical Data Management guidelines and 21 Code of Federal Regulations (CFR) Part 11 regulations on clinical data management systems for use in clinical trials. Data entry will be done via secure web interface with range and logic checks used to ensure data quality.

Fully encrypted password protected computers and/or mobile devices, for example, tablets will be used for data entry or paper forms where data cannot be entered directly into the eCRF. Management and maintenance of computers will lie with the clinical teams at the study site with remote support from central study team based at KEMRI.

The database will be kept in a locked server-room. Only the system administrators have direct access to the server and back-ups and passwords are required for access to the system and database.

All data entered into the eCRFs have identifiers that identifying the user who entered the data with the exact time and date. Retrospective alterations of data in the database are recorded in an inbuilt audit function. Time, table, data field and altered value, and the person are recorded. Logical queries developed using R-scripts will be run on the database to check for missing data or entry errors. Identified errors will be resolved by the study team.

Back-ups of the whole system including the database will be run internally several times per day. Back-ups will be stored in a secure location. Paper records will be kept in locked cabinets at the study sites.

Source documentation will be held securely at the participating study sites. Access will be granted to monitors responsible for quality assurance, for data entry staff and for purposes of medical care. Access will also be granted for audit by statutory authorities and others. Non-study team members will not be granted access. Qualified staff will supervise data collection and entry on a regular basis.

### Confidentiality {27}

Paper based clinical records will be kept in locked cabinets in the participating facilities and subsequently at KEMRI- CGMR-Coast. All electronic data, including immunological data will be kept in anonymized databases linked by a unique participant identification number to clinical data.

After the trial ends and statistical analysis is conducted, a clean final database(s) of de-identified individual-level clinical, laboratory and safety data will be produced by KEMRI-CGMR-Coast. This will be available on request for further analysis by other researchers.

### Plans for collection, laboratory evaluation and storage of biological specimens for genetic or molecular analysis in this trial/future use {33}

Of the 10ml venous blood drawn from each patient for each time point, 0.5ml of the blood sample will be used to conduct a full blood count. The remaining sample will be used for whole blood transcriptome analysis and to obtain plasma and peripheral blood mononuclear cells (PBMCs) for immunology work.

A series of cellular and serological assays will be used to characterize the immunological and metabolic status of the participants. RNA will be purified, and libraries constructed and sequenced using the Novagene platform available at a collaborating laboratory in Kenya. The sequence data will be analyzed in R. EdgeR ( https://doi.org/10.1093/bioinformatics/btp616) will be used to determine genes differentially expressed between those who died and those who recovered following treatment with or without steroid administration to determine biomarkers predictive of the outcome of the treatment. The differentially expressed genes will also be used to determine, bioinformatically, before treatment immunological status associated with the outcome of the trial. The transcriptome profile from before, during and after treatment will also be compared to determine genes up- or down-regulated following treatment and the calculated fold change will be related to the outcome and used to understand the immunological response correlated with the outcome of the trial.

The stored PBMCs will be used to phenotype in detail the cellular immune status in the trial arms before, during and after treatment using flow cytometry. Specifically, the frequency of B-cells, CD4, CD8, Natural killer (NK) cells, gamma delta ( **γ** T cells) will be described. The CD4 T cell will be further categorized into Th1, Th2, Th17 as determined by expression of IFN- **γ**, IL-4 and IL-17 through intracellular staining and flow cytometry. The cellular immune phenotype data along with blood count data will be correlated to the outcome of the trial and will facilitate interpretation of the whole blood transcriptome data obtained above. This data will be integrated with the phenotypic and transcriptomic data to understand immune and biological pathways associated with the outcome of the trial.

The stored plasma will be used to characterize, at proteomic and metabolic level, the global immune and metabolic status before treatment and changes induced following treatment. Luminex platform will be used to quantify specific cytokines using commercial kits such as the 34-plex human ProcartaPlex from Thermo Fisher Scientific that contain multiple targets that have been associated with Pneumonia including SARS-CoV-2 related pneumonia. We will also quantify a range of growth factors such as Insulin-like growth factor 1 (IGF-1) ^25^, IGF binding proteins such as IGFBP1-6 and GDF-15 ^26^ that have been shown to influence host survival under adverse conditions. 

In addition to the targeted approach, untargeted mass spectrometry approach will be used, a facility that is available at KEMRI-CGMRC. Using this approach, we identified upregulation of IGF-1 binding protein 2 (IGFBP2) that functionally inhibits IGF- and inflammation ^27^ to be associated with the risk of early death in sick malnourished children. Both before and after treatment samples for each participant will be subject to targeted and untargeted analysis and analysed as described for the whole transcriptome data to identify protein/metabolite biomarker(s) predictive of the outcome of the trial and the immunological and metabolic processes relevant to the outcome. Samples will be stored for future use according to KEMRI guidelines and consent for this will be sought from the participants sampled.

Samples will be stored for future use according to KEMRI guidelines and consent for this will be sought from the participants sampled.

## Statistical methods

### Statistical methods for primary and secondary outcomes {20a}

The primary analysis will be an intention to treat analysis in which we will compare outcomes between all patients randomized to the two study arms, irrespective of whether they receive their allocated treatment. For the primary outcome of 30-day mortality, we will use the unadjusted hazard ratio determined from Cox regression to estimate the mortality rate ratio between the treatment groups. If we detect any imbalances in potential confounders at baseline (such as age, sex, severity of illness, study site), then an adjusted Cox model will be used. If losses to follow-up do occur, patients will be censored at time of last assessment. Kaplan-Meier survival curves will be constructed to display cumulative mortality over the 30-day follow-up period.

In the per-protocol analysis we will exclude patients stopping or pausing the study medication at any time point and those withdrawn from the trial.

Complete case analyses will be used for secondary endpoints. For all secondary endpoints, we will calculate unadjusted and adjusted estimates of the effect size and corresponding 95% confidence intervals using linear, logistic, or cox proportional hazards regression as appropriate. All analysis will be done using R (Version 4.4.2, R Foundation for Statistical Computing: Vienna, Austria)

For the immunology outcome, omics data such as proteomic and transcriptomic data generated will be processed in the R programming language as described in section 33. To find genes/proteins whose mean response has significantly changed over the first 72 hours, the processed data will be fitted in a multinomial mixed model predicting the possible outcomes, adjusting for relevant baseline characteristics. The expected outcomes are, survived versus died in the treatment and placebo arms.

To understand the biological response associated with survival, induced/repressed by the treatment, functional analysis of the significant genes/proteins will be explored using R packages such as Clusterprofiller, GSVA and fgsea.

Integrative approaches will be used on the cellular phenotyping, proteomic, transcriptomic, metabolomic data to elucidate the biological processes promoted/repressed by corticosteroid treatment that are associated with survival that could be targeted with additional intervention

### Interim analyses {21b}

An interim analysis will be conducted after half of the anticipated sample has been enrolled and completed their final visit. Statistical considerations for ending the trial prior to reaching the target enrollment will be based on the Haybittle-Peto stopping rules for the primary outcome ^28^.

The analysis will explore the primary outcome i.e., to determine the effect of adjunctive corticosteroids on all-cause mortality at 30 days after randomization in both the intention-to-treat (ITT) and per protocol (PP) participants. Cox regression will be used to test for differences in the rates of the primary outcome by randomization arm. The proportional hazards assumption will be evaluated graphically and using formal statistical tests. Kaplan-Meier survival curves will be constructed to display cumulative mortality over the 30-day follow-up period.

The results of this analysis will be shared with the trial’s Data Safety Monitoring Board (DSMB) and Trial Steering Committee (TSC) who will advise on continuation of the trial based on clear survival benefits or futility.

### Methods for additional analyses (e.g. subgroup analyses) {20b}

Secondary analyses will be conducted as for the primary analysis but adjusting for the confounding factors of gender, hospital location, age at randomization and any other confounder as deemed appropriate. Although randomization into the study is not stratified by any of the baseline characteristics, stratified analyses will be presented if there is significant evidence of interaction by any of the baseline variables.

The safety analysis will be based on the safety population, that is, all participants who received a study treatment.

### Methods in analysis to handle protocol non-adherence and any statistical methods to handle missing data {20c}

During the data collection process, efforts will be made to minimize missing data occurrence. Should missing data occur, the numbers (with percentages) of losses to follow-up (defaulters and withdrawals) will be reported by randomized group at each assessment time point. If possible reasons why the data are missing will also be reported. Missing data will not be imputed in anyway.

The primary analysis will be based on an intention-to-treat (ITT) population. An unadjusted analysis of the primary outcome will also be performed on the per-protocol (PP) population. This will exclude patients stopping or pausing the study medication at any time point and those withdrawn from the trial.

### Plans to give access to the full protocol, participant level-data and statistical code {31c}

A clean de-identified database will be provided as publicly available (‘open access’) or upon reasonable request, after the final study publication is published, as per the relevant medical journal regulations. Anonymised data from the study will be shared upon request. Request for use of the study data will be assessed by the KEMRI-CGMRC Data Governance Committee to safeguard interests of participants and their communities.

## Oversight and monitoring

### Composition of the coordinating center and trial steering committee {5d}

An independent trial steering committee (TSC) has been convened for the trial. The TSC advise on the protocol, trial’s implementation including review of timeliness and recruitment and, any interim analyses to be conducted. The TSC meets quarterly during the trial duration. 

The trial has a coordination team that includes the co-principal investigators, a research medical officer, a trial coordinator and a data manager. The coordination team manages the trial’s day-to-day running, conduct safety monitoring and ensure ethical standards are maintained during the trial.

### Composition of the data monitoring committee, its role and reporting structure {21a}

An independent Data and Safety Monitoring Board (DSMB) has been convened for the trial. The main aim of the DSMB is to ensure safety of the trial’s participants through regular review of trial safety data. DSMB reports are shared with the trial investigators, the TSC and sponsor. The DSMB has five members including one chair. The membership includes a diverse representation of experts, including, specialists in clinical trials, physicians, and statisticians. The trial’s DSMB charter further details the role of the DSMB, its membership, frequency of meetings and reporting structure.

### Adverse event reporting and harms {22}

The corticosteroids to be used in this clinical trial have been used for many decades and are not expected to result in any serious unexpected adverse reactions. Adverse events (AEs) and serious adverse events (SAEs) will be defined in accordance with the International Conference on Harmonization (ICH) Guidelines for Good Clinical Practice.

Trial staff and hospital staff will review participant health status daily while in-patient and solicit for expected adverse events. Patients will be advised verbally and in writing (study information sheet) that they should inform hospital staff if they develop any change in their medical condition during their hospitalization and after discharge. Patients will in addition be given the phone number of the local trial clinical officers.

Any AE will be evaluated for seriousness, relatedness, severity, and expectedness by the study clinician. Where further medical attention is needed, the study team will organise referral and emergency admission to the nearest health facility.

All AEs that are not serious will not be reported individually but will be recorded in the CRF and summarised in an annual safety report.

All SAEs will be reported individually using an SAE form including an assessment of relatedness, severity, and expectedness.

The principal investigator (PI) or designee will be responsible for safety monitoring and reporting SAEs to the DSMB, Sponsor, ethics committees and regulatory authorities as appropriate. Those that are life-threatening, serious and unexpected or suspected to be causally related to study medication will be reported to the DSMB, Sponsor and product manufacturers.

### Frequency and plans for auditing trial conduct {23}

Trial monitoring will be conducted by independent staff from the clinical trials facility (CTF) at the KEMRI-CGMRC in Kilifi. The focus of monitoring will be on those factors critical to quality (i.e. the safety of the participants and the reliability of trial results). The frequency of monitoring will be decided by the monitoring team. Monitoring reports will be shared with the sponsor and regulator during annual reports.

### Plans for communicating important protocol amendments to relevant parties (e.g. trial participants, ethical committees) {25}

All protocol amendments will be discussed with the investigators and site leads. They will then be subjected to ethical and regulatory review before adoption.

The trial’s DSMB and TSC will be made aware of any changes to the protocol and associated forms.

## Dissemination plans {31a}

The results of the trial will be reported in a clinical study report generated by the sponsor, containing CRF data, laboratory data and safety data. The sponsor will register and disclose the existence of the results of the clinical trial on an international clinical trials registry in accordance with good practice. Individual participant identifiers will not be used in any publication of results.

Results will be published in an open access format, consistent with Good Publication Practices, the International Committee of Medical Journal Editors guidelines and funder requirements. The primary trial results will be published including all investigators meeting ICJME criteria as authors. Local investigators will take prominent roles in the primary trial results write-up. Secondary analyses will also be developed as part of KEMRI-CGMRC’s commitment to capacity development to ensure that co-investigators have additional opportunities for individual scientific output.

Results will be disseminated to the Kenya Ministry of Health, the Kenya Medical Association, all of the participating hospitals and other relevant stakeholders e.g., the WHO. Results will also be disseminated locally in the areas where the study was conducted and participants will be invited to meetings to receive feedback on the trial outcomes.

### Ethics approval and consent to participate {24}

Kenya Medical Research Institute - Scientific Ethics Review Unit (KEMRI-SERU) - SERU 4319

Pharmacy and Poisons Board of Kenya (PPB) – PPB/ECCT 21/11/02/2022(103)

Oxford tropical Research Ethics Committee (OxTREC) – OxTREC 4–22

National Commission for Science, Technology and Innovation (NACOSTI) – NACOSTI/P/22/16486

Written, informed consent to participate will be obtained from all participants or their legally authorized representatives.

Deferred written informed consent will be sought for patients too unwell to provide consent within 48 hours of admission if written assent is provided by their legally authorized representatives.

### Consent for publication {32}

The investigators are willing to provide a model consent form on request

## Discussion

Despite community acquired pneumonia being a major cause of mortality among adults in low- and middle-income countries, very few clinical trials that are relevant to this setting have been conducted. The SONIA trial is a pragmatic randomized clinical trial that aims to determine whether the addition of corticosteroids to standard of care in patients with community-acquired pneumonia reduces mortality. The trial is being conducted in public hospitals in Kenya, many of which have not participated in research activities before. However as has been argued previously, in order to generate results that are widely applicable to the real world, clinical trials need to be as simple as possible ^29^. The pragmatic nature of the SONIA trial makes it easy to conduct it in the course of routine clinical work without exerting too much extra work on staff at the participating hospitals, while at the same time aiming to generate reliable results that are relevant to the setting. The clinical question being addressed is highly relevant to the patients, eligibility criteria are simple, the intervention readily available, and follow up procedures easy to implement.

## Study status

Current Protocol – Version 2.0 dated 01 February 2022

Recruitment start date – 25 April 2022

Actual recruitment end date – 30 June 2024

## Abbreviations

AE                    Adverse Event

ATP                  According-to-protocol

CAP                 Community Acquired Pneumonia

CFR                  Code of Federal Regulations

CI                     Confidence Interval

CME                 Continuous Medical Education

COVID-19        Corona Virus Disease 2019

CRF                  Case Report Form

CTF                  Clinical Trials Facility

DSMB              Data and Safety Monitoring Board

ERC                 Ethics Review Committee

GCP                 Good Clinical Practice

ICF                  Informed Consent Form

ICH                  International Conference on Harmonization

IgG                  Immunoglobulin G

IRB                  Institutional Review Board

ISF                   Investigator site file

ITT                  Intention to treat

IU                    International Units

KEMRI            Kenya Medical Research Institute

LAR                 Legally authorized representative

LMIC                Low- and Middle-Income Countries

NRA                 National regulatory authority

OxTREC           Oxford Tropical Research Ethics Committee

PI                      Principal Investigator

PIS                    Patient information sheet

PP                      Per Protocol

SAE                    Serious Adverse Event

SAP                    Statistical Analysis Plan

SERU                  Scientific Ethics Review Unit

SOP                    Standard operating procedure

SUSAR               Suspected unexpected serious adverse reaction

TSC                    Trial Steering Committee

WHO                   World Health Organization

## Data Availability

A clean de-identified database will be provided as publicly available (‘open access’) or upon reasonable request, after the final study publication is published, as per the relevant medical journal regulations. Information collected or generated during this study may be anonymised for use to support health policies and/or further research. Any further research using information from this study must first be approved by a local or national expert committee to make sure that the interests of participants and their communities are protected. The protocol’s SPIRIT checklist and flow diagram are available at https://doi.org/10.5281/zenodo.7180419
