# Supplementary material for: A pragmatic randomized controlled trial of standard care versus corticosteroids plus standard care for treatment of pneumonia in adults admitted to Kenyan hospitals (SONIA)
Source: Wellcome Open Res. 2025 May 28;7:269. Originally published 2022 Oct 27. [Version 2] doi: 10.12688/wellcomeopenres.18401.2 (PMC12177450; doi:10.12688/wellcomeopenres.18401.2)
Supplement: Peer review history

# Reviewer report: Reviewer response for version 2

**DOI:** 10.21956/wellcomeopenres.26398.r125341

**Published:** 2025-07-04

**Authors:** Naveed Saleem

**Affiliations:** 1 School of Clinical Medicine, University of New South Wales Medicine & Health, Sydney, Australia, Australia

I appreciate you inviting me to examine this publication, which describes a clinical trial design that tackles the crucial topic of whether corticosteroids can lower death rates among adults in a sub-Saharan African environment who have community-acquired pneumonia.

 The proposed trial is adequately and thoroughly described in the manuscript. Since this trial has been approved and is now accepting participants, I have mostly restricted my comments to offering clarifications regarding a few queries in this manuscript.

 The SONIA trial has addressed a critical question with high potential impact in an understudied population. The rationale is strong, the pragmatic design is well-suited to the context, and the primary outcome and methods are generally clear. However, the following few concerns may be addressed, which might enhance the validity of the findings.

- There must be a validated CAP severity score (e.g., CURB-65) in the inclusion criteria documentation and baseline data collection. Pre-specify subgroup analysis by severity.
- Try to provide a detailed, recommended antibiotic regimen (specific drugs, doses, frequencies, durations, IV->oral switch criteria) in an appendix.
- There is a need to define and mandate prospective recording of key co-interventions (antibiotics, oxygen, ICU admission) and use objective criteria for SAE assessment/attribution. Ensure DSMB monitors for differential management.
- Table 1 lists bioequivalent doses but doesn't specify if tapering is allowed after the 10-day course. Abrupt cessation after 10 days could theoretically cause issues, though the risk at these doses/durations is low. There is a need to state explicitly if tapering is prohibited, permitted at clinician discretion, or standardised.

Is the study design appropriate for the research question?

Yes

Is the rationale for, and objectives of, the study clearly described?

Yes

Are sufficient details of the methods provided to allow replication by others?

Yes

Are the datasets clearly presented in a useable and accessible format?

Partly

Reviewer Expertise:

Nanomedicine, Medicinal Chemistry, Microbiology, antibiotics resistance

I confirm that I have read this submission and believe that I have an appropriate level of expertise to confirm that it is of an acceptable scientific standard.

# Reviewer report: Reviewer response for version 2

**DOI:** 10.21956/wellcomeopenres.26398.r124505

**Published:** 2025-06-18

**Authors:** Matthias Briel, Christof Schönenberger

**Affiliations:** 1 Division of Clinical Epidemiology, University Hospital Basel, Basel, Basel-Stadt, Switzerland; 2 Department of Clinical Research, University Hospital Basel, University of Basel, Basel, Switzerland

Dear Dr. Lucinde, dear SONIA trial team, 

 Thank you very much for the response and the incorporation of my suggestions. I have no further comments.

Is the study design appropriate for the research question?

Yes

Is the rationale for, and objectives of, the study clearly described?

Yes

Are sufficient details of the methods provided to allow replication by others?

Yes

Are the datasets clearly presented in a useable and accessible format?

Not applicable

Reviewer Expertise:

I am a medical doctor and methodologist with a focus on clinical trials

We confirm that we have read this submission and believe that we have an appropriate level of expertise to confirm that it is of an acceptable scientific standard.

# Reviewer report: Reviewer response for version 1

**DOI:** 10.21956/wellcomeopenres.20400.r79397

**Published:** 2024-06-11

**Authors:** Matthias Briel, Christof Schönenberger

**Affiliations:** 1 Department of Clinical Research, University Hospital Basel, University of Basel, Basel, Switzerland; 2 Division of Clinical Epidemiology, University Hospital Basel, Basel, Basel-Stadt, Switzerland

I would like to thank the author for the opportunity to review their RCT protocol on corticosteroid in patients with CAP in Kenya. I have already dealt with this topic for some review projects and therefore hope to be able to make a constructive contribution to improving the protocol.

 Overall I think the protocol is well reported. I listed some minor points below:

1. I was not able to find the registry number.
2. In the abstract, I would provide some more concrete information.
  1. In what kind of CAP patients are you interested in? Hospitalized CAP patients right (mild or severe)?
  2. What do you mean by clinical diagnosis of CAP?
  3. What is standard of care for CAP?
  4. Immunologic sub study is to prominent in abstract
3. In the introduction, the authors have succeeded in showing why the existing evidence is difficult to transfer to the setting in Kenya. However, I noticed the following points:
  1. Maybe you could shorten the first paragraph from introduction section, since it is well known that CAP is a major problem. For example, you could leave out the unpublished data.
  2. The references in the part about pathophysiology of CAP are very old. I would recommend adding some newer paper on the dysregulation of immune system and potential role of steroids in the modulation of the immune system.
  3. Not all the meta-analysis you cited were able to show benefit of steroids regarding survival, specially not the newest one you included from Briel. et al. 2018[Ref 1]. Please correct that paragraph.
  4. Maybe for the result publication worth to consider: Since the meta-analysis from Briel in 2018[Ref 1], there are some important new trials, for example NCT02517489 which was able to show survival benefit of steroids in ICU CAP patients. I think you should include the newest evidence from trials in the background section.
  5. When you say steroids are not part of current guidelines, I would mention the respective guidelines. USA? Kenya?
  6. Please try to summarize the evidence of steroids in COVID better. I would not say RECOVERY is a “recent” study. Also, I would try to explore what are potential similarities or differences between CAP and COVID and why do you think steroids could also be effective in CAP? See also background and discussion in NCT02517489 result paper.
4. How do you plan to assess time to death? Is that objective not a bit redundant to death after 7, 14, 21, 30 days?
5. Which system will be used for the eCRF?
6. The following exclusion criteria are all a bit similar to me:
  1. Patients who in the opinion of the attending clinician, require to be treated with steroids.
  2. Known or suspected condition which in the opinion of attending clinician requires treatment with steroids, including but not limited to chronic obstructive pulmonary disease, asthma, adrenal insufficiency, Pneumocystis Jirovecii pneumonia (PCP).
  3. If the clinician strongly suspects COVID-19 and wants to provide steroids to the patient because of this suspicion, then the patient will be excluded from the trial.
7. Describe the test used for the sample size calculation.
8. Do you plan to stratify the randomization for trial center?
9. Which subsample of PBMC will be subjected to metabolomic analysis?
10. I would suggest the authors to use an adjusted cox model for the primary outcome regardless of baseline imbalance. Give the target sample size, major baseline imbalance is highly unlikely and if present, suspicious. However, there is evidence suggesting to adjust trial analysis for co-variates, see for example: Lee PH et a;, 2016 [Ref 2]  or Hauck WW et al,1998 [Ref 3]
11. Please specify the “established methods” you are using in R for the omics data. (In the same paragraph, you talk about a placebo arm instead of control arm)
12. Are all analysis, also for the clinical endpoints, conducted with R? Please specify this.
13. Please specify any considerations regarding multiple testing for the interim analysis and subgroup analysis.

Is the study design appropriate for the research question?

Yes

Is the rationale for, and objectives of, the study clearly described?

Yes

Are sufficient details of the methods provided to allow replication by others?

Yes

Are the datasets clearly presented in a useable and accessible format?

Not applicable

Reviewer Expertise:

I am a medical doctor and methodologist with a focus on clinical trials

We confirm that we have read this submission and believe that we have an appropriate level of expertise to confirm that it is of an acceptable scientific standard, however we have significant reservations, as outlined above.

## References

[1] : Corticosteroids in Patients Hospitalized With Community-Acquired Pneumonia: Systematic Review and Individual Patient Data Metaanalysis. *Clin Infect Dis* .2018;66(3) : 10.1093/cid/cix801 346-354 10.1093/cid/cix801 29020323

[2] : Covariate adjustments in randomized controlled trials increased study power and reduced biasedness of effect size estimation. *J Clin Epidemiol* .2016;76: 10.1016/j.jclinepi.2016.02.004 137-46 10.1016/j.jclinepi.2016.02.004 26921693

[3] : Should we adjust for covariates in nonlinear regression analyses of randomized trials?. *Control Clin Trials* .1998;19(3) : 10.1016/s0197-2456(97)00147-5 249-56 10.1016/s0197-2456(97)00147-5 9620808

# Response

**Published:** 2025-04-04

**Authors:** Ruth Lucinde

**Affiliations:** KEMRI-Wellcome Trust Research Programme, Kenya

We would like to express our thanks for your time and your comments on this article.  Below are our responses. Where needed, revisions to the text of the article have been made. 

1. I was not able to find the registry number.

Apologies, these are provided in the revised article. The trial is registered with the Pan-African Clinical Trials Registry (PACTR202111481740832) and the ISRCTRN registry (ISRCTN36138594).

1. In the abstract, I would provide some more concrete information.
  1. In what kind of CAP patients are you interested in? Hospitalized CAP patients right (mild or severe)?
  2. What do you mean by clinical diagnosis of CAP?
  3. What is standard of care for CAP?
  4. Immunologic sub study is too prominent in abstract

Thank you. We will revise the abstract section to reflect the following responses to comments shared:

1. The trial will determine the effect of corticosteroids in participants who comprise all patients hospitalized with CAP. As testing for disease severity is limited in our trial sites, we will not limit enrollment by disease severity.

 

1. This refers to diagnosis based on the WHO recommended diagnosis of CAP based on clinical features (World Health Organization ( 2012), https://iris.who.int/handle/10665/77751 and World Health Organization (‎2022)‎ https://iris.who.int/handle/10665/365237).  We will not use radiological or laboratory tests to confirm the diagnosis of CAP.

 

1. The standard care for CAP in Kenya is combination therapy with a beta-lactam and macrolide antibiotic ( https://healthsciences.uonbi.ac.ke/sites/default/files/2023-06/KNH%20Empiric%20Antimicrobial%20Therapy%20Guidelines.pdf).

This is explained further in the article’s main text.

1. We agree and will remove mention of the immunologic sub-study in the revised article’ abstract.

1. In the introduction, the authors have succeeded in showing why the existing evidence is difficult to transfer to the setting in Kenya. However, I noticed the following points:
  1. Maybe you could shorten the first paragraph from introduction section, since it is well known that CAP is a major problem. For example, you could leave out the unpublished data.

This has been done in the revised article

1.
  1. The references in the part about pathophysiology of CAP are very old. I would recommend adding some newer paper on the dysregulation of immune system and potential role of steroids in the modulation of the immune system.

Thank you. We have updated the manuscript with more recent references.

1.
  1. Not all the meta-analysis you cited were able to show benefit of steroids regarding survival, specially not the newest one you included from Briel. et al. 2018[Ref 1]. Please correct that paragraph.

Thank you, we have revised this paragraph in the revised article

1.
  1. Maybe for the result publication worth to consider: Since the meta-analysis from Briel in 2018[Ref 1], there are some important new trials, for example NCT02517489 which was able to show survival benefit of steroids in ICU CAP patients. I think you should include the newest evidence from trials in the background section.

Indeed, there have been newer studies and meta-analysis reporting survival benefits of corticosteroids. We have updated the references in the revised article to represent this.

1.
  1. When you say steroids are not part of current guidelines, I would mention the respective guidelines. USA? Kenya?

We will specify that at the time of protocol development, corticosteroids were not recommended in the management of community acquired pneumonia in Kenyan and WHO guidelines

1.
  1. Please try to summarize the evidence of steroids in COVID better. I would not say RECOVERY is a “recent” study. Also, I would try to explore what are potential similarities or differences between CAP and COVID and why do you think steroids could also be effective in CAP? See also background and discussion in NCT02517489 result paper.

We have discussed this further in the revised article’s background section. At the time of protocol development, late 2021, RECOVERY was a recent trial, and the CAPE COD trial’s results had not been published.

1. How do you plan to assess time to death? Is that objective not a bit redundant to death after 7, 14, 21, 30 days?

We agree. Because time to death analysis is essentially covered by the survival analysis to be conducted for the primary objective, we will compare the proportion of participants dying at 7-, 14- ad 21- days post-enrolment by trial arm as part of the secondary analyses. We will amend this statement in the revised protocol

1. Which system will be used for the eCRF?

We will use REDCaP (Research Electronic Data Capture), a secure, web-based software platform, designed to support data capture for research studies and have now mentioned this in the revised article.

1. The following exclusion criteria are all a bit similar to me:
  1. Patients who in the opinion of the attending clinician, require to be treated with steroids.
  2. Known or suspected condition which in the opinion of attending clinician requires treatment with steroids, including but not limited to chronic obstructive pulmonary disease, asthma, adrenal insufficiency, Pneumocystis Jirovecii pneumonia (PCP).
  3. If the clinician strongly suspects COVID-19 and wants to provide steroids to the patient because of this suspicion, then the patient will be excluded from the trial.

We agree. We separated these for ease of understanding for the reviewers and trial team at trial sites. We have revised them into one statement in the revised article

1. Describe the test used for the sample size calculation.

We used this formula for a two independent sample study with a dichotomous endpoint (incidence of death by trial arm) from Rosner B. *Fundamentals of Biostatistics*. 7th ed. Boston, MA: Brooks/Cole; 2011. Using the parameters that we entered into the formula we obtain a required sample size of 2070 which we then increased by 5.3% to allow for losses to follow up. 

 p _1_, p _2_ = incidence rates for groups #1 and #2

 Δ = |p _2_-p _1_| = absolute difference between two incidence rates

 n _1_ = sample size for group #1

 n _2_ = sample size for group #2

 α = probability of type I error (0.05)

 β = probability of type II error (0.15)

 z = critical Z value for a given α or β

 K = ratio of sample size for group #2 to group #1    

1. Do you plan to stratify the randomization for trial center?

Yes, we will.

1. Which subsample of PBMC will be subjected to metabolomic analysis?

Apologies. We have realized this section was included erroneously and have now deleted the section

1. I would suggest the authors to use an adjusted cox model for the primary outcome regardless of baseline imbalance. Give the target sample size, major baseline imbalance is highly unlikely and if present, suspicious. However, there is evidence suggesting to adjust trial analysis for co-variates, see for example: Lee PH et al;, 2016 [Ref 2]  or Hauck WW et al,1998 [Ref 3]

Thank you for the suggestion. This will be incorporated in the SAP as a secondary analysis.

1. Please specify the “established methods” you are using in R for the omics data. (In the same paragraph, you talk about a placebo arm instead of control arm)

We have revised the article to refer to the control arm and added information on the specific R-package to be used for the RNAseq data and included a reference.

1. Are all analysis, also for the clinical endpoints, conducted with R? Please specify this.

Yes, they are. We have clarified this in the revised article

1. Please specify any considerations regarding multiple testing for the interim analysis and subgroup analysis.

We used the Haybittle-Peto ( https://doi.org/10.1038/bjc.1976.220 and https://doi.org/10.1259/0007-1285-44-526-793 ) rules for the single interim analysis that we conducted. These guidelines have strict criteria (p<0.001) to guard against premature stoppage of the trial while also minimizing alpha spending so that conventional thresholds can be used at the final analysis. The trial is not powered to assess subgroup effects and we will not adjust for multiple testing for these analyses. We will state in the final report that the subgroup results are purely of an exploratory nature and not hypotheses tests.

# Reviewer report: Reviewer response for version 1

**DOI:** 10.21956/wellcomeopenres.20400.r56741

**Published:** 2023-05-26

**Authors:** Stephen J Aston

**Affiliations:** 1 University of Liverpool, Liverpool, England, UK

Many thanks for asking me to review this manuscript describing a clinical trial protocol that addresses the important question of the efficacy of corticosteroids in reducing mortality amongst patients with community-acquired pneumonia in adults in a sub-Saharan African setting.

 The manuscript describes the proposed trial clearly and with sufficient detail. As this trial is approved and currently open to recruitment, I have largely limited my comments to points of clarification with respect to the description of the trial in this manuscript, or where relevant, additional justification of trial design choices made.

1. Trial registration - Is the trial registered with an international trial registry? Please could the authors provide details.
2. Use of multiple corticosteroids - I appreciate that this a pragmatic study and that the authors have included some mitigation against drug stockouts, but I think that it would be helpful to provide further justification of the decision to permit use of different corticosteroids. Do the published meta-analyses of corticosteroids in the treatment of CAP, suggest any differences in the efficacy or safety of different corticosteroids?
3. Discussion about potential impact of TB - Observational studies of CAP from Kenya (Scott *et al.,* Lancet 2000 ^1^) and other settings in sub-Saharan Africa (Aston *et al.* AJRCCM 2019 ^2^) suggest that in contrast to the high-income settings where previous clinical trials of corticosteroids in CAP have largely been conducted, a significant proportion of patients recruited to this trial will have tuberculosis - either as a primary reason of presentation or as a co-pathogen. The authors hint at this with reference to a previous trial in South Africa (ref 14), but do not discuss further the potential implications and/or proposed mitigations. Whilst not mandated by protocol, do they expect to have fairly complete testing for TB by Xpert or otherwise to permit subgroup analysis?  
4. Discontinuation and modification of intervention - In this pragmatic and open-label study, the authors have allowed for the possibility that clinicians may choose to stop steroids in the intervention group or start them in the control group. To what extent do they anticipate that this may happen and what is the anticipated impact on the power of the trial to detect a mortality difference between arms?
5. Capture of adverse events - The authors highlight that steroids are commonly used drugs with well characterised safety profiles and mention in the introduction the harmful effects of long-term use. There are well recognised harms associated with short term use of steroids (e.g. hyperglycaemia, gastrointestinal bleed, delirium). Could the authors describe how confident they are that the proposed methods for recording adverse events will capture these potential effects of steroids and, in particular, do so without bias given the open-label nature of the study?  
6. Non-mortality endpoints - Given the pragmatic, open-label nature of the trial, the authors have opted for a primary endpoint of mortality. Previous clinical trials of steroids in CAP have used alternative endpoints including clinical/treatment failure, ICU admission/mechanical ventilation, length of stay, readmission to hospital. Will the trial capture data to be able to report these endpoints in exploratory analyses? 
7. Impact of HIV - Based on previous observational data, what proportion of recruits do the authors anticipate will be HIV-infected? Whilst not mandated by protocol, do the authors anticipate that HIV testing will be fairly comprehensive, such that they will be able to do appropriate sub-group/multivariable/interaction analyses?
8. Severity of illness - In the statistical methods section, the authors indicate that adjusted Cox model could be developed that takes into account severity of illness. Could they elaborate on how severity of illness will be characterised? For example, in peripheral sites, will physiological observations including saturations be reliably captured?

There are a couple of additional important references in this area that I would suggest should be included:

 Saleem N, Kulkarni A, Snow TAC, Ambler G, Singer M, Arulkumaran N. Effect of Corticosteroids on Mortality and Clinical Cure in Community-Acquired Pneumonia: A Systematic Review, Meta-analysis, and Meta-regression of Randomized Control Trials. Chest. 2023 Mar;163(3):484-497. doi: 10.1016/j.chest.2022.08.2229. Epub 2022 Sep 7. PMID: 36087797 ^3^.

 Torres A, Sibila O, Ferrer M, Polverino E, Menendez R, Mensa J, Gabarrús A, Sellarés J, Restrepo MI, Anzueto A, Niederman MS, Agustí C. Effect of corticosteroids on treatment failure among hospitalized patients with severe community-acquired pneumonia and high inflammatory response: a randomized clinical trial. JAMA. 2015 Feb 17;313(7):677-86. doi: 10.1001/jama.2015.88. PMID: 25688779 ^4^.

Is the study design appropriate for the research question?

Yes

Is the rationale for, and objectives of, the study clearly described?

Yes

Are sufficient details of the methods provided to allow replication by others?

Yes

Are the datasets clearly presented in a useable and accessible format?

Not applicable

Reviewer Expertise:

Clinical infectious diseases in particular respiratory tract infection.

I confirm that I have read this submission and believe that I have an appropriate level of expertise to confirm that it is of an acceptable scientific standard.

## References

[1] : Aetiology, outcome, and risk factors for mortality among adults with acute pneumonia in Kenya. *The Lancet* .2000;355(9211) : 10.1016/S0140-6736(00)02089-4 1225-1230 10.1016/S0140-6736(00)02089-4 10770305

[2] : Etiology and Risk Factors for Mortality in an Adult Community-acquired Pneumonia Cohort in Malawi. *Am J Respir Crit Care Med* .2019;200(3) : 10.1164/rccm.201807-1333OC 359-369 10.1164/rccm.201807-1333OC 30625278 PMC6680311

[3] : Effect of Corticosteroids on Mortality and Clinical Cure in Community-Acquired Pneumonia: A Systematic Review, Meta-analysis, and Meta-regression of Randomized Control Trials. *Chest* .2023;163(3) : 10.1016/j.chest.2022.08.2229 484-497 10.1016/j.chest.2022.08.2229 36087797 PMC10808071

[4] : Effect of corticosteroids on treatment failure among hospitalized patients with severe community-acquired pneumonia and high inflammatory response: a randomized clinical trial. *JAMA* .2015;313(7) : 10.1001/jama.2015.88 677-86 10.1001/jama.2015.88 25688779

# Response

**Published:** 2025-04-04

**Authors:** Ruth Lucinde

**Affiliations:** KEMRI-Wellcome Trust Research Programme, Kenya

We would like to express our thanks for your time and your comments on this article.  Below are our responses. Where needed, revisions to the text of the article have been made. 

 Trial registration - Is the trial registered with an international trial registry? Please could the authors provide details. Yes, the trial is registered with the Pan-African Clinical Trials Registry (PACTR202111481740832) and the ISRCTRN registry (ISRCTN36138594).

 We have included this in the revised article Use of multiple corticosteroids - I appreciate that this a pragmatic study and that the authors have included some mitigation against drug stockouts, but I think that it would be helpful to provide further justification of the decision to permit use of different corticosteroids. Do the published meta-analyses of corticosteroids in the treatment of CAP, suggest any differences in the efficacy or safety of different corticosteroids?

 While data on the subgroup effect of different corticosteroids have not been consistent in published meta-analyses of corticosteroids in the treatment of CAP (Jheng-Yan et al.,2023 and Pitre et al., 2023) our decision to allow multiple corticosteroids was largely informed by the variable availability of low-dose oral corticosteroids at the study sites.  

 Maintaining a pragmatic approach allows for our trial to generate real-world evidence on the effect of corticosteroids. Our trial is not powered to detect differences in effect by corticosteroid but may provide insights that may inform future studies evaluating these differences. Discussion about potential impact of TB - Observational studies of CAP from Kenya (Scott et al., Lancet 20001) and other settings in sub-Saharan Africa (Aston et al. AJRCCM 20192) suggest that in contrast to the high-income settings where previous clinical trials of corticosteroids in CAP have largely been conducted, a significant proportion of patients recruited to this trial will have tuberculosis - either as a primary reason of presentation or as a co-pathogen. The authors hint at this with reference to a previous trial in South Africa (ref 14), but do not discuss further the potential implications and/or proposed mitigations. Whilst not mandated by protocol, do they expect to have fairly complete testing for TB by Xpert or otherwise to permit subgroup analysis?  Testing for pulmonary tuberculosis is largely available in Kenya but underutilized (Oliwa et al., 2018, https://doi.org/10.1186/s12879-018-3237-z ). Given the pragmatic nature of the trial, we will rely on clinician-initiated tests for pulmonary tuberculosis to define the proportion of participants with co-infection at enrollment. We however don’t expect this pragmatic approach to provide enough numbers for subgroup analyses as it is dependent on access to, availability of and clinician requests for these tests. Discontinuation and modification of intervention - In this pragmatic and open-label study, the authors have allowed for the possibility that clinicians may choose to stop steroids in the intervention group or start them in the control group. To what extent do they anticipate that this may happen and what is the anticipated impact on the power of the trial to detect a mortality difference between arms? With continued engagement and sensitization on trial activities during the conduct of the trial, we hope to limit these instances. Additionally, we will adhere to a strict inclusion and exclusion criteria to limit the possibility of enrolling participants who are likely to then have steroids included or withdrawn from their management. Our sample size calculations allow for 5% drop-off ( ~109 participants) and we anticipate this will cover any instances of clinician-initiated protocol deviations that lead to withdrawals. Capture of adverse events - The authors highlight that steroids are commonly used drugs with well characterised safety profiles and mention in the introduction the harmful effects of long-term use. There are well recognised harms associated with short term use of steroids (e.g. hyperglycaemia, gastrointestinal bleed, delirium). Could the authors describe how confident they are that the proposed methods for recording adverse events will capture these potential effects of steroids and, in particular, do so without bias given the open-label nature of the study?  By recording vital signs, measuring and recording random blood sugars for all participants and soliciting for all common side effects of corticosteroids, we believe we’ll identify and record most instances of corticosteroid-related adverse events while our patients are in-patient. Our calls to participants at 14- and 30- days post-enrolment are also intended to solicit for adverse events in addition to recording of vital status. We however recognize that some adverse events may be unreported and thus don’t expect our summary of safety data to be used in place of hypothesis testing Non-mortality endpoints - Given the pragmatic, open-label nature of the trial, the authors have opted for a primary endpoint of mortality. Previous clinical trials of steroids in CAP have used alternative endpoints including clinical/treatment failure, ICU admission/mechanical ventilation, length of stay, readmission to hospital. Will the trial capture data to be able to report these endpoints in exploratory analyses? Yes, the trial will record data on length of stay and report the effect of steroids on this in exploratory analyses. On the other hand, ICU admission and mechanical ventilation are largely inaccessible to many in Kenya and tracking readmission to hospital may be difficult if these readmissions are to non-trial sites. We therefore may not have enough data for exploratory analyses of these alternative endpoints. Impact of HIV - Based on previous observational data, what proportion of recruits do the authors anticipate will be HIV-infected? Whilst not mandated by protocol, do the authors anticipate that HIV testing will be fairly comprehensive, such that they will be able to do appropriate sub-group/multivariable/interaction analyses? As with testing for pulmonary tuberculosis, testing for HIV is largely available but depended on clinician requests and patient consent ( https://nacc.or.ke/wp-content/uploads/2018/11/HIV-estimates-report-Kenya-20182.pdf ). The prevalence of HIV among patients presenting to these trial sites is expected to be ~ 5% as we report elsewhere (Lucinde et al., 2024, https://doi.org/10.1186/s12879-024-10140-6 ). However, we do not expect the proportion of patients having these test done during their admission to be enough to conduct subgroup analyses. Severity of illness - In the statistical methods section, the authors indicate that adjusted Cox model could be developed that takes into account severity of illness. Could they elaborate on how severity of illness will be characterised? For example, in peripheral sites, will physiological observations including saturations be reliably captured? We will use oxygen saturation at enrollment as a surrogate measure of disease severity for subgroup analyses. The trial team will ensure oxygen saturation is measured for all participants at enrolment and during the duration of their hospital stay. The final analyses for the trial will be outlined in the trial’s statistical analysis plan (SAP) and will reflect whether the trial will deem this approach to quantifying disease severity as adequate or not. Discussions with the trial’s steering committee (TSC) and data safety and monitoring board (DSMB) will guide the development of the final SAP. There are a couple of additional important references in this area that I would suggest should be included: Thank you for these suggestions. These references have been included in the revised article
